# Acute Kidney Outreach to Reduce Deterioration and Death (AKORDD) trial: the protocol for a large pilot study

**DOI:** 10.1136/bmjopen-2016-012253

**Published:** 2016-08-19

**Authors:** Tarek Samy Abdelaziz, Antje Lindenmeyer, Jyoti Baharani, Hema Mistry, Alice Sitch, R Mark Temple, Gavin Perkins, Mark Thomas

**Affiliations:** 1 Department of Renal Medicine, Birmingham Heartlands Hospital, Heart of England NHS Foundation Trust, Birmingham, UK; 2 Institute of Applied Health Research, University of Birmingham, Birmingham, UK; 3 Division of Health Sciences, Warwick Medical School, University of Warwick, Birmingham, UK; 4 Department of Epidemiology, Biostatistics and Public Health, University of Birmingham, Birmingham, UK; 5 Department of Critical Care, Birmingham Heartlands Hospital, Heart of England NHS Foundation Trust, Birmingham, UK

**Keywords:** Acute kidney injury, hospital acquired, Acute kidney injury, community acquired, Electronic alerts, Rapid response teams, Outreach, Healthcare outcomes

## Abstract

**Introduction:**

Acute kidney injury (AKI) contributes to morbidity and mortality, and its care is often suboptimal and/or delayed. The Acute Kidney Outreach to Reduce Deterioration and Death (AKORDD) study is a large pilot testing provision of early specialist advice, to improve outcomes for patients with AKI.

**Methods and analysis:**

This before and after study will test an Outreach service for adult patients with AKI, identified using the national algorithm. During the 2-month before phase, AKI outcomes (30-day mortality, need for dialysis or AKI stage deterioration) will be observed in the intervention and control hospitals and their respective community areas; no interventions will be delivered. Patients will receive good standard care. During the 5-month after phase, the intervention will be delivered to patients with AKI in the intervention hospital and its area. Patients with AKI in the control hospital and its area will continue to have good standard care only. Patients already on dialysis and at end of life will be excluded. The interventions will be initially delivered via a phone call, with or without a visit to the primary clinician, aiming at rapidly establishing the aetiology, correcting reversible causes and conducting further appropriate investigation. Surviving stage 3 patients will be followed-up in an AKI clinic. We will conduct qualitative research using focus group-based discussions with primary and secondary care clinicians during the early and late phases of the trial. This will help break down potential barriers and improve care delivery.

**Ethics and dissemination:**

Patients will be contacted about the study allowing them to ‘opt out’. The work of an Outreach team, guided by AKI alerts and delivering timely advice to clinicians, may improve outcomes. If the results suggest that benefits are delivered by an AKI Outreach team, this study will lead to a full cluster randomised trial.

**Trial registration number:**

NCT02398682: Pre-results.

**Strengths and limitations of this study**

- Acute Kidney Outreach to Reduce Deterioration and Death (AKORDD) is a large pilot study and the first controlled trial in unselected acute kidney injury (AKI) in the UK.
- It employs a before and after design in control and intervention hospitals and their areas.
- It uses the national AKI algorithm in hospital and community to identify cases.
- The intervention is delivered by the Outreach team for all eligible cases in working hours.
- With only two sites, it is not a full cluster randomised study.

## Background and rationale

Acute kidney injury (AKI) is a common condition. Its prevalence in UK is estimated to be >20% of emergency admissions.^1^ Worldwide incidence is about 21.6% in adults in hospital settings, as shown in a recent meta-analysis.^2^ Mortality due to AKI is high. Recent studies show an overall mortality of >23% in the UK,^3^ and a similar percentage worldwide.^2^ There are recognised deficiencies in the clinical care of patients with AKI.^4^ The UK's National Confidential Enquiry into Patient Outcome and Death (NCEPOD)^4^ showed that 14% of fatal AKI cases were avoidable. One large UK study found that mortality in patients with AKI was significantly higher in the 55% of acute trusts that did not have onsite renal teams.^5^ AKI aetiology is diverse, and it usually occurs in the setting of other comorbidities. However, few studies have looked into the effect of non-renal comorbidities on outcome. Charlson comorbidities have been used to predict outcome in end-stage renal disease.^6–8^ Our previous work examined the role of comorbidity in AKI, demonstrating the impact of solid and haematological malignancies, as well as the total burden of non-malignant comorbidities.^9^ Intensive care patients with AKI and uncontrolled malignancy are known to have poor outcome.^10^

Advances in technology show promise in the early identification of AKI, using electronic alerts.^9^ ^11^ Theoretically, bringing the recent rise in creatinine to clinicians' attention should prompt improvements in management. However, a recent study using alerts alone failed to demonstrate any improvements in outcome.^12^

The concept of an Outreach team has been established in critical care for many years, offering rapid assessment to deteriorating patients. One large cluster randomised trial (CRT) failed to show a significant impact of Medical Emergency Team in reducing hospital cardiac arrests.^13^ In the UK, the introduction of critical care Outreach in an 800-bed general hospital significantly reduced mortality.^14^ Two large meta-analyses were conducted analysing trials of rapid response teams (RRTs). Outreach teams were successful in reducing non-intensive care unit cardiac arrest by 34%, but mortality was not significantly reduced.^15^ Overcoming the barriers to implementation of the RRT service would further improve outcome.^16^ The dissociation between decline of cardiac arrest rates and mortality is not fully understood.

The use of an Outreach team for renal patients is novel. We piloted a renal Outreach team in 2009, offering early specialist advice for patients who developed AKI. The team delivered limited dose (3 hours a day, 5 days/week) telephone advice to clinicians looking after patients with AKI. Two hundred and sixty-two alerts were identified with a successful telephone call in 88% of the cases. Considerable numbers of recommendations were made. These included optimisation of fluid status and medication management.^17^ There has also been one other, smaller pilot of early intervention in AKI.^18^

The adverse sequelae of an AKI episode continue even after its complete or partial resolution. It may be valuable to have early renal follow-up, especially for severe cases. A retrospective study suggested that early follow-up of patients with AKI, especially those not previously known to nephrology services, may reduce mortality.^19^

The true cost of AKI is unknown as existing studies are retrospective. Calculating the cost of AKI syndrome is important to assess the burden to the healthcare systems. The healthcare costs for patients with AKI have a robust relationship to the severity of AKI.^20^ It was estimated in previous studies that prevention of 30% of AKI cases would save the National Health Service (NHS) between £130 and £186 million per year.^21^ In a European study, it was shown that conservative management of AKI, including the initial hospitalisation and 2-year follow-up, incurred average costs of €34 000, and this increased to €40 000–€55 000 if a patient had dialysis (depending on the modality).^22^ The financial burden includes inpatient, critical care and postdischarge costs (the latter encompass progression of chronic kidney disease (CKD) with or without renal replacement therapy).^21^

Overall, the study's primary aim is to test the feasibility, systems for and value of the Outreach team approach on the measured outcomes in patients with AKI, before a planned CRT.

## Methods

### Overall study design

We will run a quasi-experimental trial of a complex multifactorial intervention.^23^ Using a before and after study design, we will pilot the Outreach service to patients with AKI. Patients will be recruited once they have an electronic alert indicating that they have AKI. Inclusion and exclusion criteria will then be applied (box 1). There will be no patient-level randomisation to avoid the risk of contamination, as this is a pilot for a CRT. The study has been approved by (1) the National Research Ethics Service (Reference: 14/EM/0184, NRES Committee East Midlands—Nottingham 1, approved 22 May 2014) and (2) the Confidentiality Advisory Group (CAG Reference: CAG 2-07(a)/2014—approved 30 May 2014).

Box 1 **Inclusion and exclusion criteria for the Acute Kidney Outreach to Reduce Deterioration and Death (AKORDD) study**

Inclusion criteria:

- Adult (≥18 years) patients with an alert due to AKI from a blood test arising from Heartlands or Good Hope Hospitals (and their associated postcodes for outpatients/primary care).
- AKI stage 1, 2 or 3.

Exclusion criteria:

- Patient deceased at the time of Outreach team intervention.
- Patient already accepted for dialysis by HEFT renal unit.
- Known dialysis/end-stage renal disease patients (excluded from alerts).
- Other patients with stable chronic kidney disease (definition of stable CKD), or progressive CKD where there is no obvious change in the rate of progression of the CKD (ie, no acute deterioration, just steady progression).

Patients with CKD and stage 1 AKI based on an absolute creatinine rise of ≥26 µmol/L but who have a relative rise of ≤15% in creatinine are regarded as stable CKD and therefore not eligible for the study.

- Children or young people <18 years of age.
- Patients in the terminal phase of malignancy or end-stage major organ disease where they have little potential to benefit from Outreach. These would typically be identified as having a life expectancy likely to be <3 months.
- Patients lacking mental capacity (unless enrolment agreed in writing with a personal consultee or nominated consultee (after phase) or personal consultee (before phase)).
- Other technical aspects of making alert not credible: inadequate data; suppressed creatinine at baseline.

It was impractical to take written informed consent from the large numbers of patients involved over two hospitals, when the study was focused on providing AKI Outreach to clinicians. Therefore, it was agreed with the research ethics committee that we would write to all enrolled patients giving them an ‘opportunity to dissent’ or ‘opt out’ of the study. The opt out letter will be sent to the patient's home address as soon as possible after enrolment. In conjunction with the opt out letter, a leaflet explaining AKI will be sent to patients in the intervention group only.

**Figure 1:**
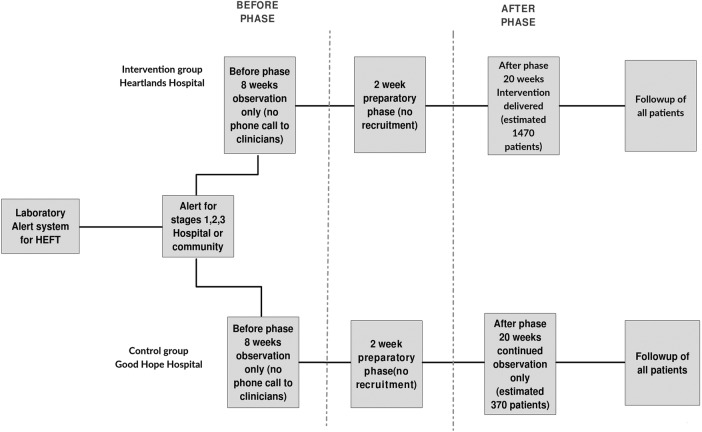
Study flow chart.

The study is registered at ClinicalTrials.gov (NCT02398682). It is funded by the Research for Patient Benefit programme of the UK National Institute for Healthcare Research (PB-PG-1111-26038) and sponsored by the Heart of England Foundation Trust. The trial has two phases and four groups (2 during each phase—see also figure 1):

- The before phase
  During the 2-month before phase, patients in the two groups will be observed for the outcome measures, without any intervention, thus defining outcomes with good standard care. There will be two before phase groups:
  1. Heartlands and area patients—patients with AKI in secondary and primary care—observed only.
  2. Good Hope and area patients—patients with AKI in secondary and primary care—observed only.
- The after phase
  During the 5-month after phase, the intervention will be delivered to the intervention group (Heartlands and area) only. There will be two after phase groups:
  1. Heartlands hospital and area patients—intervention provided for patients with AKI in secondary and primary care, mainly using Outreach via telephone (see below).
  2. Good Hope hospital and area patients—patients with AKI in secondary and primary care—observed only.

### Data collection and recruitment

Data collection will be carried out using a Concerto clinical portal (V.8.3) and database (Orion Health, London, UK), embedded within the Trust's IT system. All AKI alerts (discussed below) are visible to the study team in a list in Concerto. Patients not previously enrolled in the study can be virtually ‘transferred’ into the study database at enrolment.

A key point is that each patient can only be recruited once to the study, and thus a given patient cannot be recruited to the before phase, and then later to the after phase. This avoids the ethical issue of double recruitment. Thus, a patient enrolled during the before phase cannot be enrolled in the after phase, if they have a further episode of AKI. The recruitment of more ‘recurrent’ patients with AKI during the 8-week before phase presents a potential limitation, but in our view, it is balanced by the much longer 20-week after phase.

Patients enrolled in the intervention (Heartlands after phase) group remain a ‘customer’ in the care of the AKI Outreach team if they have a recurrent episode of AKI, and their clinician(s) can receive further Outreach intervention(s) as required. In practice, we believe that this is the way any AKI Outreach team will work in future. The study recruitment will run from mid-2015 to later in 2016.

### Core study methodology

To allow replication of the model throughout the wider NHS, we provide the updated TIDieR checklist (see online supplementary files).^24^

10.1136/bmjopen-2016-012253.supp2 Supplementary file

#### Alerts

The electronic AKI alerts are the trigger for the afferent arm of the intervention. The alerts are generated within the laboratory software (Telepath, Computer Sciences Corporation) according to the national algorithm.^25^ It compares the current patient creatinine to a value in the past (baseline creatinine). The staging follows KDIGO guidelines for AKI,^26^ where 0 is no AKI and 3 is the most severe stage. Alerts are made visible to all users of the electronic patient records, with the stage being stated as part of all urea, creatinine and electrolyte reports. The alerts from our hospital laboratory also contain a hyperlink to online guidance, with separate guidance for hospital and community alerts. Alerts are not issued for patients in the dialysis units and renal wards. We refer to the current location of the patient when discussing community (primary care) or hospital alerts. Obviously some of the hospital alerts will represent what was community-acquired AKI, but Outreach focuses on the current location of the patient.

#### Hospital sites

The Heart of England Foundation Trust consists of three hospitals. Good Hope Hospital was selected as control site as it is larger and relatively similar to the intervention site of Heartlands Hospital in its services. For a detailed description of both hospitals, please refer to online supplementary file 1.

10.1136/bmjopen-2016-012253.supp1 Supplementary file

#### Primary care sites

A large proportion of all AKI cases begin in the community. We, therefore, felt that it was important to include patients with AKI in the community in the study, as this provides the earliest opportunity to intervene in the care of the patient. Primary care clinicians see AKI alerts in the patients' electronic laboratory reports (implemented after consultation as part of the start of the study—about 9 months ahead of the national schedule). We have mapped >17 000 emergency admissions presenting to the three hospitals during a calendar month to identify which areas predominantly present to Heartlands hospital, and similarly to Good Hope hospital. We are, therefore, able to allocate any patient with AKI in the community in these postcodes to either the control (postcode areas B23, B24, B35, B43, B44 and B72–B79) or the intervention arms (postcode areas B7–B11, B25, B26, B33, B34, B36, B37, B40 and B46). A small number of general practices in our area do not use the hospital laboratories of the Trust, and therefore, any alerts for their patients were not available to the study team.

### After phase sample size

For the before phase, no sample size is required and eligible patients during the study period will be recruited. The sample size was calculated with the intent of estimating uncertainty of outcomes, to inform the design of the future cluster trial. Our previous work suggested that the combined outcome would be seen in about 40% of patients having an alert. With a sample size of >1000 (intervention group—Heartlands and area, after phase), a 95% CI of width 6% (37% to 43%) can be calculated. A sample size of 370 (control group—Good Hope and area, after phase) will allow us to produce a 95% CI of width 10% (35% to 45%).

### Study team

The AKI Outreach team will function during working hours, 5 days/week. The ideal service, addressing AKI 7 days a week, is beyond the resources of this pilot. The model is a mixed skills team, allocating tasks using an electronic list of alerts and the electronic study database. The core Outreach team consists of an experienced renal consultant (usually the Chief Investigator), renal research fellow (working at junior specialist registrar level) and a critical care research (trained in AKI care) nurse. The core clinical team members will be primarily responsible for delivering the interventions, in the form of telephone consultation, with or without a ward visit, triggered by the AKI alert. The core team members are supported by other research staff, who help with the research data collection. Each working day patients are prioritised according to AKI severity.

### Data collected

In the study database, we will collect data on the causes of AKI and the associated comorbidities for all patients, as well as medication used at the time of AKI. Causes will be determined in pragmatic fashion from the electronic records, including prerenal/renal hypoperfusion, nephrotoxic medications, intrinsic or specialist renal diseases (not covered elsewhere), urological/postrenal and surgical causes. We will determine comorbidities according to a modified Charlson scheme as previously reported,^9^ and in addition stage solid neoplasia using the Surveillance, Epidemiology, and End Results staging system.^27^ ^28^

### Interventions

In majority of cases, interventions will be in the form of phone call to the clinical team looking after the patient. The phone call will be structured to obtain verbal consent of the clinician at the start of the call. The duration of the call will be tailored according to clinical complexity and expected outcome of the case. All interventions will be recorded on the trial database in order to measure ‘dose’. In view of the large numbers managed by a small Outreach team, we anticipate that most interventions will rely on a single call, with no follow-up. Key feasibility measures in the intervention phase will be the time from alert to intervention, and the median number of recommendations (in comparison to our previous work^17^).

### Intervention package

The Outreach team will advise on an evidence-based package of care (these interventions are limited to the intervention arm):

1. *Diagnostic and monitoring recommendations*—to rapidly establish a credible diagnosis of the cause of AKI including:
  - Improved assessment of volume status;
  - Standardised use of urine dipstick (often missed);
  - Appropriate sepsis investigations;
  - Urgent ultrasound with suspected obstruction, while avoiding early ultrasound in patients at low risk of obstruction.
2. *AKI treatment recommendations*—to treat correctable causes of AKI:
  - Rapid, limited treatment of hypovolaemia, with avoidance of iatrogenic fluid overload, recently recognised as a significant cause of mortality in AKI;
  - Rapid sepsis therapy;
  - Cessation of potentially nephrotoxic agents (many well known, such as ACE inhibitors and non-steroidal drugs, but others less well known such as Aciclovir);
  - Urgent relief of urinary tract obstruction.
3. *Recommendations to manage the acute illness:*
  - Where needed, recommendations will be made to address key aspects of the major underlying comorbidity;
  - Improve nutrition, as malnutrition is known to be associated with poor outcome. Dietetic referral will be made for all appropriate inpatients with stage 3 AKI.
4. *Pathway recommendations:*
  - For inpatients, refer to the Renal and/or Critical Care Outreach teams, as appropriate;
  - For patients in the community, arrange urgent or immediate outpatient review or admission, as appropriate;
  - Arrange formal palliative care when necessary.
5. *Stage-based AKI management:*
  - Stage 1 patients will typically receive a single intervention call;
  - Stage 2 patients will usually receive a single intervention call and be discussed with the renal consultant;
  - Stage 3 inpatients will be reviewed by a consultant, ideally within 24 hours of the AKI Outreach team being aware of their presence in the intervention hospital (Heartlands Hospital);
  - Rapid AKI follow-up clinic—we will offer surviving patients with stage 3 AKI a rapid follow-up clinic appointment, ideally within 7 days of discharge, or within 7 days of the alert if the patient is not admitted to hospital.

### The health economics substudy

We will pilot a prospective health economic assessment, calculating the cost of AKI. We aim to define with more accuracy the true total cost of an AKI episode. We will also pilot assessment of the costs of the Outreach service and its recommendations, using national tariffs for the latter. The information will be extracted from the electronic patient records or the paper records. The costs of long-term sequelae such as dialysis will be included. For the purpose of the pilot health economics substudy, we will obtain a sample of 50 patients, divided into 25 patients from the intervention group and 25 from the control group. We will open ‘recruitment weeks’ to run simultaneously at both sites until the numbers are met at each site. We will break down the service components (including, eg, the time of the phone call), any laboratory or radiological tests, and we will use the NHS cost codes. We will use the EQ-5D-5L questionnaire,^29^ which is a standardised short quality-of-life questionnaire, which has been used in various populations, including critically ill patients with AKI,^30^ since its development in 1990.^31^ We will record the five dimensions of health status and the visual analogue scale results,^32^ at the time of AKI (0 months). The EQ-5D-5L, and in addition a health resource use questionnaire (based on those from DIRUM (Database of Instruments for Resource Use Measurement)),^33^ will be completed by telephone at 3, 6, 9 and 12 months.

### Fidelity

We will pilot a fidelity assessment, examining intervention adherence, in conjunction with the health economic analysis, using the 25 patients recruited to the above analysis at the intervention site. In the fidelity and health economic work, each patient record will be examined in detail to extract the adherence to recommendations as well as all the costs of the admission.

### Quantitative analysis of clinical data

#### Estimates for the CRT

The main aim of this study is to test feasibility of the intervention and all study procedures. In addition, this pilot study will allow estimates of the uncertainty of the primary outcome (deterioration or death) to be used in planning a cluster trial. Secondarily, data from this study can be used to assess the prognostic model previously introduced. In addition, simple analyses will be conducted to look at CKD progression or development of end-stage renal disease as a result of AKI episode, and survival to discharge to a more dependent setting than at admission.

### Outcomes

The primary purpose of this study is to assess feasibility and inform a full cluster randomised study. The primary outcome for the full study, also collected in this study, is reflected in the trial acronym: deterioration or death of the patient with AKI, and is a combination of:

- All-cause death within 30 days;
- Any need for dialysis or renal replacement therapy (intermittent haemodialysis (typically at the Renal Unit of Birmingham Heartlands Hospital) or continuous renal replacement therapy (typically in the intensive care units of the Trust)) within 30 days;
- Progression of AKI stage without dialysis within 30 days after the alert (stage progression is stage 1 deteriorating to 2 or 3; stage 2 deteriorating to stage 3).

These were chosen to reflect the major, potentially modifiable, outcomes of concern for patients (death and/or dialysis) and renal units or hospitals (death, dialysis or deterioration). Stage progression (compared to non-progression) is known to be associated with increased mortality and length of stay in a multivariate analysis,^34^ and is a useful surrogate end point.

Secondary outcomes for the main study, collected to assess feasibility here, are as follows:

**Table:** 

Measured outcome	Time window
Percentage of patients admitted to hospital	Within 14 days of alert
Percentage requiring any critical care admission	During index admission
Other key events (see below)	During index admission
Percentage discharged to institutional care	After index admission
All-cause mortality	At 90, 182 and 365 days
Renal function (eGFR)	At 90, 182 and 365 days

In a pragmatic study, patients will not be recalled for renal function testing; at the time points above, we will use the last observation carried forward for all living patients. Key events are these events as given in the discharge letter:

1. Cause of AKI: new glomerulonephritis or new urinary obstruction diagnosis.
2. Complication of AKI not requiring dialysis: new pulmonary oedema, uraemia ≥30 mmol/L or hyperkalaemia (≥6.0 mmol/L).

### Statistical analysis

The focus of the study is to assess feasibility of all aspects prior to full-scale evaluation. The data collected in this pilot study will be used to assess the uncertainty of the primary outcome. Further analyses will separately compare the before and after arms at the intervention and control sites. We note that the intervention and control sites cannot be directly compared. The control site data will be used to assess any ‘system-wide’ change in AKI outcomes. Additionally, survival analyses will be carried out, examining time to first composite primary outcome, and Kaplan-Meier survival curves produced for each of the four groups. This pilot study is not powered to provide a full comparison of the before and after phases. Among the end points, the use of dialysis and frequency of non-dialytic AKI complications will give an indication of the safety of the intervention. We will analyse estimated Glomerular Filtration Rate (eGFR) and CKD stage at a group level and changes at individual patient level. We will also explore the potential of using these data to validate our previous prognostic index.^9^

### Qualitative work

#### Prestudy

A challenging area of development is the communication with primary care and secondary care clinicians. We carried out prestudy qualitative research on the potential role of an AKI Outreach team. The aim of the focus groups was to explore the barriers that might hinder the application of optimum care for patients with AKI. We conducted eight focus groups with the primary clinicians and hospital doctors to explore their experience with AKI management, and the best methods to enhance this. Our qualitative work will be separately presented.

#### Poststudy

The poststudy qualitative work will use focus groups and interviews to examine the experience of clinicians receiving AKI Outreach advice during the intervention phase, aided by the voice recording of the Outreach call. We will use purposeful sampling to explore a spread of possible perspectives, for example, doctor role/seniority, different reasons for AKI and different AKI stages. We will carry out a realistic evaluation of the role of Outreach, using one-to-one interviews for general practitioners and focus groups for four hospital specialties (critical care; acute medicine; elderly care and surgical specialties). The recordings for those clinicians will be evaluated using a realistic evaluation,^35^ to explore ‘what works, for whom and in what circumstances’.

### Discussion and limitations of the study

The national algorithm for staging of AKI means that identification of patients with AKI is standardised. The ‘opt out’ consent process enables large-scale recruitment. The ethos of AKI Outreach is that advice is offered to the primary clinician, who may choose to act upon or ignore that advice. A key challenge in implementing the intervention is reaching and influencing the decision maker(s) within the primary team, especially as they have varying roles and beliefs.

The AKI Outreach service runs only 40 hours/week and not out-of-hours. As this is a pilot for a full CRT, there were only two sites and randomisation was not undertaken. Inevitably, the intervention and the control sites are different to a certain extent. The latter does not have on-site nephrology services. A full CRT is needed to provide a definitive answer regarding any benefits of this approach. What would a successful pilot look like? The delivery of recommendations acceptable to clinicians, comparable to our previous work (median of 3 within a median of 15 hours), and to large numbers of patients is a key indicator of success. The control group should show no evidence of a system-wide change in before and after AKI outcomes. The intervention group will provide evidence of the feasibility and safety of the intervention.

### Conclusions

The Acute Kidney Outreach to Reduce Deterioration and Death (AKORDD) is a pilot study of an acute kidney Outreach team, and is needed to lay the foundations for use of this novel intervention in developed healthcare systems. It potentially provides a way of improving outcomes in the syndrome that is AKI. If it shows that AKI Outreach is feasible and safe, then it will act as an important ‘pathfinder’ study for a large, multicentre, CRT.
