# Supplementary material for: Acute Kidney Outreach to Reduce Deterioration and Death (AKORDD) trial: the protocol for a large pilot study
Source: BMJ Open. 2016 Aug 19;6(8):e012253. doi: 10.1136/bmjopen-2016-012253 (PMC5013506; doi:10.1136/bmjopen-2016-012253)
Supplement: Supplementary file [file bmjopen-2016-012253supp1.pdf]

Supplementary file 1

Heart of England Foundation Trust and AKORDD intervention and control areas

The Trust has approximately 1300 beds on three hospital sites, with about 250,000 emergency attendances, and about 65,000 emergency admissions (of all ages) per annum. The relevant details including number of beds on each site are given below. The smallest hospital (Solihull Hospital) in the group and its surrounding postcodes were excluded from the study. The intervention and control hospitals and their respective areas are shown below.

Birmingham Heartlands Hospital and area (intervention site)

Population served in 2011:	332000
Bed number (all ages):	665 beds
Intensive care (level 2 and 3) beds:	12 level 2† beds, 11 level 3 beds* with facility for continuous renal replacement therapy.
On site nephrologists:	Full time.
Dialysis unit:	32 station unit including 11 acute haemodialysis stations.
Emergency department:	≈ 121, 000 unplanned attendances per annum (financial year 2014/15)

Good Hope Hospital and area (control site)

Population served in 2011:	406300
Bed number (all ages):	440 beds
Intensive care (level 2 and 3) beds:	12 level 2† / 3 beds (typically 50/50% split), all with facility for continuous renal replacement therapy.
On site nephrologists:	None; visiting nephrologist for clinic and urgent referrals only.
Dialysis unit:	None.
Emergency department:	≈ 83, 000 unplanned attendances per annum (financial year 2014/15)

*Intensive care beds only (Thoracic high dependancy beds included)

†Does not include Coronary care

Population data from: http://www.ons.gov.uk/ons/about-ons/business-transparency/freedom-of-information/previous-foi-requests/people--population-and-community/population-for-every-postcode-district-in-england-and-wales/index.html

Allocation of postcodes for AKORDD
Birmingham Heartlands Hospital ● East Birmingham

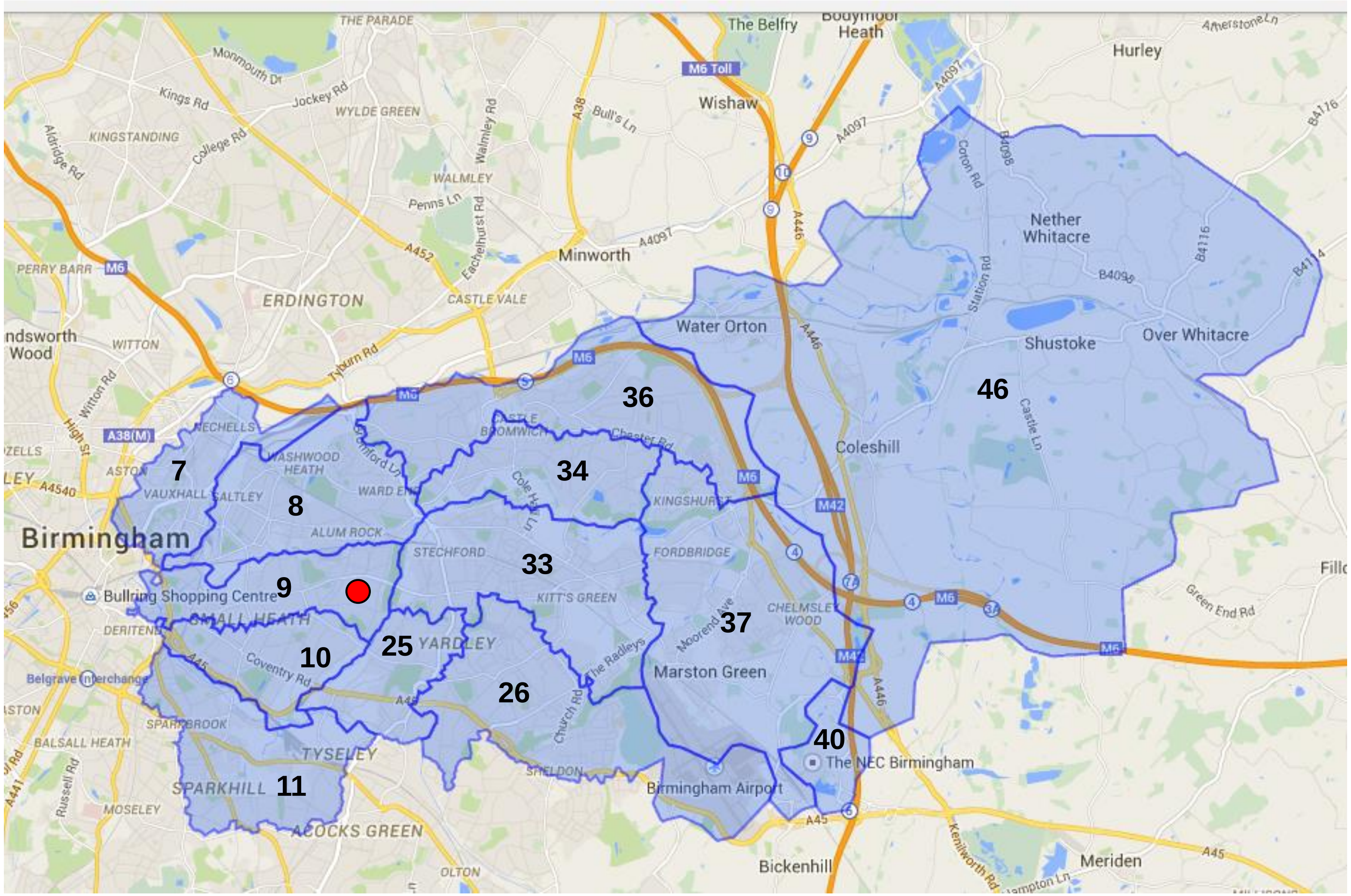

B7
B8
B9
B10
B11
B25
B26
B33
B34
B36
B37
B40
B46

Allocation of postcodes for AKORDD
Good Hope Hospital ● Northeast Birmingham

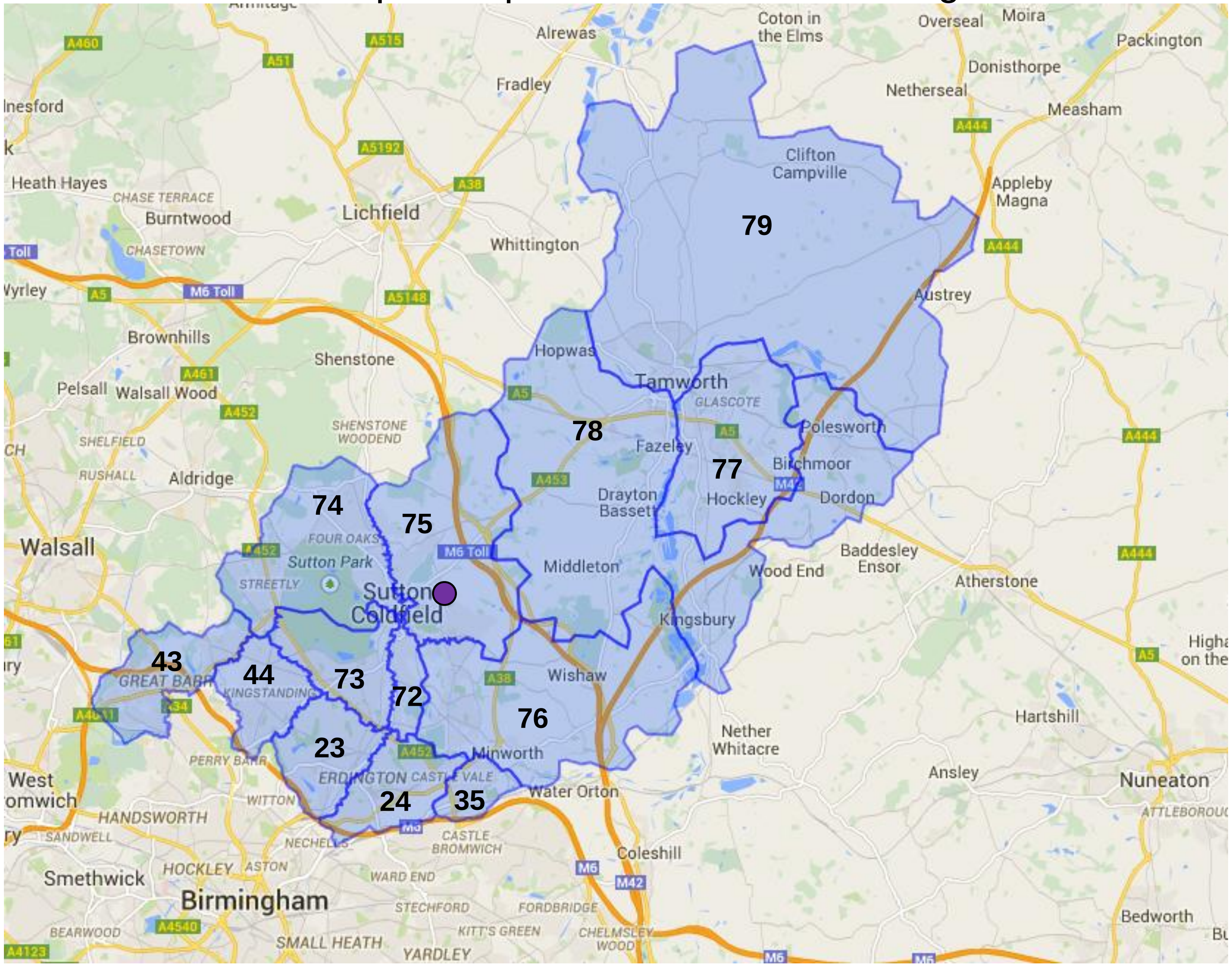

B23
B24
B35
B43
B44
B72
B73
B74
B75
B76
B77
B78
B79
